# Miniaturized Flexible Corrosion-Resistant Tag Antenna with Folding Arm Based on Graphene Film

**DOI:** 10.3390/mi17050634

**Published:** 2026-05-21

**Authors:** Meng Zeng, Xin Zhao, Hongyu Zhou, Jinling Li, Rongguo Song, Haoran Zu, Daping He

**Affiliations:** 1 Hubei Engineering Research Center of RF-Microwave Technology and Application, Wuhan University of Technology, Wuhan 430070, China; 347466@whut.edu.cn (M.Z.); xzhao@whut.edu.cn (X.Z.); zhouhongyu@whut.edu.cn (H.Z.); lijl@whut.edu.cn (J.L.); hedaping@whut.edu.cn (D.H.); 2 School of Information Engineering, Wuhan University of Technology, Wuhan 430070, China

**Keywords:** UHF frequency band, graphene-assembled film, miniaturization, flexible RFID tag, corrosion resistance

## Abstract

Radio frequency identification (RFID) technology has been widely adopted in a variety of practical applications. Usually, the size of a passive tag antenna largely determines the read performance of tag. However, excessively large tag antennas can hinder their practical application and a tag that is too small has poor performance. In this paper, a compact, flexible and corrosion-resistant folding dipole tag antenna is proposed, which has a geometrical dimension of 24 mm × 13 mm (0.074$\lambda_{0}\times 0.040\lambda_{0}$). It is designed on only one surface of a flexible polyethylene terephthalate (PET) substrate, which can be folded. The paper proposes a single-sided laser-patterned GAF/PET flexible RFID tag that is mechanically folded to form a backside dipole arm without vias, targeting compact and corrosion-resistant UHF RFID operation. Changing the size of the folding arm can effectively adjust the resonant frequency and impedance of the tag antenna. A stepped radiation arm is used to extend the current path and lower the resonance frequency. The capacitance and inductance effects introduced by loading a T match for reducing the resonant frequency of the tag to the useful UHF RFID band. Finally, it can achieve a power transfer coefficient of 99.9% and exhibit high impedance matching between the tag antenna and the chip. The proposed tag antenna uses graphene-assembled film (GAF) as its conductor material. Thanks to the physicochemical properties of GAF, the proposed tag antenna maintains stable radiation performance even after prolonged exposure to acidic (5 wt%), alkaline (5 wt%), and salt (5 wt%) corrosion, as well as more than 1000 mechanical bending cycles. When the EIRP of the reader is 2.2 W, the maximum read range of the tag in the 800–1000 MHz is 1.38 m.

## 1. Introduction

Radio frequency identification (RFID) technology has been widely adopted in a variety of practical applications, including asset tracking, warehousing and logistics, identity verification, and access control. Passive UHF tag antennas are equipped with chips that enable data transmission and storage. They offer large-capacity memory and long-range read capabilities at a low cost, making them a subject of significant interest [1]. Passive tags have no battery power and derive all the energy they need to operate from the electromagnetic waves emitted by the reader. Designing passive UHF tags involves balancing antenna gain, impedance, and antenna size. Usually, the size of a passive tag antenna largely determines the read performance of tag. Larger tag antennas typically offer farther read ranges. However, excessively large tag antennas can hinder their practical application. To miniaturize tag antennas, commonly used methods include employing substrates with high dielectric constants [2,3], enhancing capacitive and inductive effects [4,5,6] and extending the current path [7,8].

Miniaturization can be easily achieved by employing substrates with high dielectric constants. In [3], ceramic elements (ε ≈ 100) were used to design a compact tag antenna measuring 34 mm (radius) × 20 mm, which achieved an actual read range of 5 m in testing. Such substrates not only increase manufacturing costs but also introduce significant dielectric loss, ultimately resulting in reduced radiation efficiency of the tag antenna. The design principles of the planar inverted-F antenna (PIFA) are applied to tag antennas, achieving miniaturization by implementing multiple ground vias [9,10] or shorting plates [11]. The miniaturization of 18 mm × 4 mm × 2 mm was achieved by using six metal shorting vias in [12], with an actual test read range of 1.2 m. However, PIFA structures involve complex designs, and the resonant frequency is highly sensitive to via parameters, leading to cumbersome and time-consuming tuning. In order to solve this problem, a folded patch design is employed, which eliminates the need for shorting vias. Furthermore, the function of the vias is effectively transferred to the sides by establishing a shorting wall, making it easier to tune the antenna. In [13], four rotationally symmetrical planar inverted L antennas (PILAs) with a loop-fed structure were proposed. It achieved omnidirectional radiation within a 40 mm × 40 mm × 1.6 mm footprint, with a measured read range of 5.9 m on a metal surface. However, the resonant frequency of folding patch antennas in a miniaturized size is often too high, and the profile is large. Tuning mechanisms, such as slots [14,15,16] and meandered lines [17,18,19,20], are often required to lower the resonance frequency by extending the current path, thereby increasing the electrical length to reduce the physical dimensions. In meandering lines, the opposite current directions at the bends create a canceling effect. Two bent lines that are too close together will cause mutual coupling, which may lose gain and reduce the read range of the tag antenna. In [21], an interdigitated open-branch structure was employed to improve inductance and impedance matching. The device achieved dimensions of 55.2 mm × 44.2 mm × 1.5 mm, with a measured reading range of 5 m in free space. In addition, current UHF tags primarily use metal materials as conductors, which have poor corrosion resistance. This often requires additional technical measures, such as injection molding, but this inevitably causes the tag to harden and increases its size.

In this paper, a flexible, corrosion-resistant folding dipole tag antenna is proposed, which is extremely compact. It offers high adjustability and can effectively tune the tag resonant frequency to achieve −10 dB impedance bandwidth in the range of 920.6–932.4 MHz while maintaining readability within 800–1000 MHz. Inspired by the inverted-F structure, which is difficult to fabricate due to its vias and large profile, a new antenna based on the folding dipole structure is proposed. The proposed antenna is simple to manufacture, and the antenna structure is very compact. It is designed on only one surface of a flexible polyethylene terephthalate (PET) substrate, which can be folded. The tag is mechanically folded to form a backside dipole arm without vias for miniaturization. It is made to form conductor stubs connected to the back after folding to increase the capacitive inductance effect. The folded arm itself does not occupy any additional surface area of the tag antenna. The other arm of the dipole forms a stepped inverted L structure to increase the current path and reduce the resonance frequency. This effectively reduces the resonant frequency of such a small antenna to the available UHF band. In addition, the T-matching structure is used to embed the chip into the feeder without additional space. The tag structure is simple and does not require any metal vias. A variety of tuning mechanisms enable the proposed tag to achieve a wider frequency tuning range while maintaining its miniaturized design. Thanks to the physicochemical properties of graphene-assembled film (GAF), the proposed tag antenna maintains stable radiation performance even after prolonged exposure to acidic (5 wt%), alkaline (5 wt%), and salt (5 wt%) corrosion, as well as more than 1000 mechanical bending cycles. These results verify its excellent corrosion resistance and mechanical stability. The content of this paper is organized as follows: Section 2 discusses the conductor materials and structure of the tag antenna. Section 3 describes the antenna design, analyzes the antenna radiation using the characteristic mode, and presents the test results for the tag antenna. The tag performed well after undergoing corrosion and bending, with minimal variation in read range.

## 2. Materials and Methods

### 2.1. Properties of GAF

In the design of RF and microwave devices, the conductivity of conductor materials exerts a decisive influence on signal-transmission performance. However, enhancing the conductivity of materials suitable for microwave applications while simultaneously maintaining their mechanical stability remains challenging. In recent years, GAF has attracted considerable attention in the RF field owing to its high electrical conductivity, chemical stability, and environmental compatibility [22].

**Figure 1:**
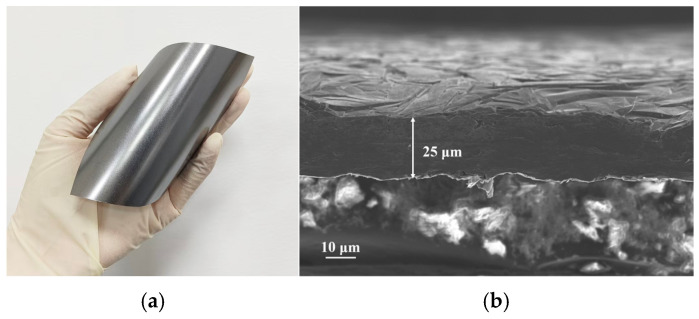
Properties of GAF: (**a**) The digital photo of GAF; (**b**) The cross-section SEM image of GAF.

The proposed tag antenna uses GAF as its conductor material. The physical and electrical properties of the GAF used in this work have been quantitatively assessed based on our previous foundational study [23]. The specific GAF exhibits an electrical conductivity of 1.1 × 10^6^ S/m and low sheet resistance of 3.5 × 10^−2^ Ω/sq. The conductivity of GAF is lower than that of copper 5.8 × 10^6^ S/m. However, existing studies have demonstrated that when the electrical conductivity of GAF exceeds the critical threshold of 10^6^ S/m, it satisfies the performance requirements for RF and microwave devices. The digital photograph of GAF shown in Figure 1a illustrates its smooth and dense surface, which exhibits a uniform metallic luster. It also exhibits excellent mechanical flexibility and good self-supporting properties. This indicates that it is not easy to break under deformation and offers excellent processability, which is very suitable for flexible electronic device design. The cross-sectional SEM image in Figure 1b clearly shows the distinct interface of the film. Its surface appears to be rough in microscale. Its interior presents a dense layered stacked structure with uniform orientation and tight layers, showing no signs of delamination. This microstructure, which is assembled in an orderly manner from graphene, is the structural basis for its macroscopic mechanical flexibility and gives the material excellent electrical conductivity potential. The GAF has a total thickness of approximately 25 ± 1 µm and is uniformly laminated on PET (ε_r_ = 3.3, tanδ = 0.003 at 10 GHz) substrate with low dielectric loss and excellent mechanical flexibility.

### 2.2. Tag Antenna Configuration

**Figure 2:**
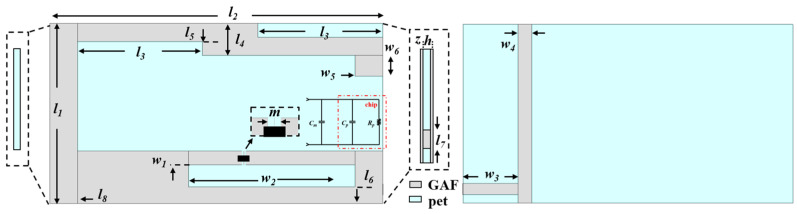
Structure of GAF tag antenna.

The proposed tag antenna structure is shown in Figure 2. Using a laser engraving machine (Protolaser U4, LPKF, Hanover, Germany), the shape of the tag antenna is engraved on the GAF. These GAF were pre-deposited on flexible PET film with a thickness of h = 0.08 mm. The tag consists of three parts: a stepped inverted-L dipole radiation arm, a T-matched feeding structure and the other arm of the dipole folded to the back.

The radiating arm of the top dipole is bent to evolve into a stepped structure. This stepped bending structure is actually a method of slightly disturbing and extending the surface current path. It can make the surface current flow along the edge of the step in a meandering manner without increasing the X-Y plane area of the antenna, thereby increasing the effective electrical length of the antenna. Then, a T-match structure composed of a feed loop and a part of the main radiation arm is introduced in parallel. First, it acts as an impedance converter to adjust the width and spacing of T-shaped branches, which can change the current distribution ratio. Second, the T match structure itself is equivalent to a parallel inductor, which can generate inductive reactance to achieve impedance matching with the chip. The Impinj Monza R6 chip is connected via a narrow gap (*m*). The chip has a read sensitivity of −20 dBm and an input impedance of 11.73–j118.06 Ω at a frequency of 927 MHz. According to the electromagnetic field mirror theory, if a metal ground surface is placed on the back of a dielectric plate, the mirror current of the same amplitude and in the opposite direction will be excited on the ground. This current comes from the alternating current generated by the top radiation arm. Because the PET substrate thickness of this tag antenna is very thin, the radiation field between the ground plane and the radiation arm will be strongly interfered with. This will have a serious impact on antenna radiation. In order to prevent the above from happening, a design with a shortened ground plane was adopted. Physically speaking, this is equivalent to folding the other arm of a traditional half-wave dipole to the back of the antenna, so that the bottom conductor layer no longer acts as a reflector. It will be demonstrated later that the folding arm is directly involved in radiation. The size parameters of the tag antenna are shown in Table 1.

## 3. Results and Discussion

### 3.1. Design of the Tag Antenna

**Figure 3:**
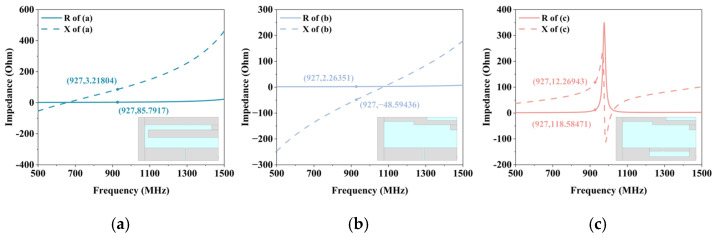
Evolution of antenna structure and corresponding impedance curves: (**a**) The impedance of folding dipole preliminary antenna with uniform curved structure; (**b**) The impedance of folding dipole antenna with stepped structure; (**c**) The impedance of final tag antenna with stepped and T-match structure.

To investigate the characteristics of the antenna, the structural design of the tag antenna is described. In the preliminary design of the tag antenna, the folding dipole basic antenna model shown in Figure 3a is first constructed. The prototype antenna uses a conventional uniform bending structure to extend the current path, and the impedance frequency curve shows that the resonant frequency of the antenna is not in the UHF. Such a structure cannot be matched with chip conjugation in the UHF. Moreover, the geometry of the prototype is simple, and it lacks enough parameter variables for independent fine-tuning of impedance. Even after adjusting these parameters, it is difficult to achieve impedance matching with multiple chips.

**Figure A1:**
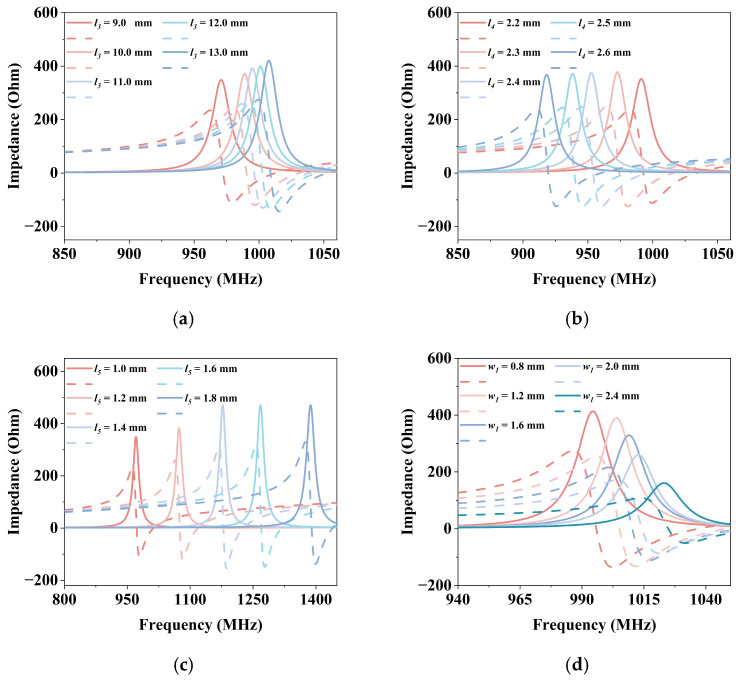

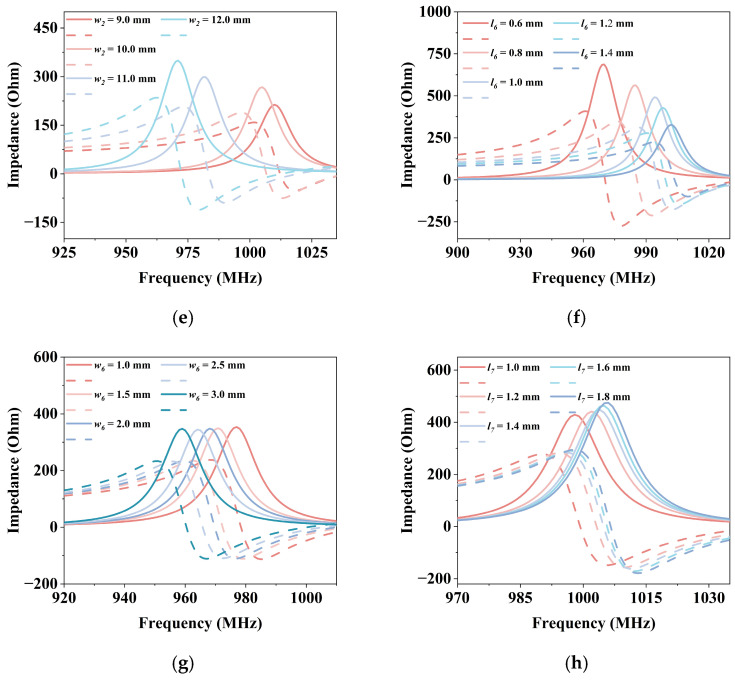
Parametric analysis of the stepped structure and T match branch: (**a**) Effects of varying the parameter of the stepped structure *l*_3_; (**b**) Effects of varying the parameter of the stepped structure *l*_4_; (**c**) Effects of varying the parameter of the stepped structure *l*_5_; (**d**) Effects of varying the width of the T match *w*_1_; (**e**) Effects of varying the length of the T match *w*_2_; (**f**) Effects of varying the parameter of the T match *l*_6_; (**g**) Effects of varying the parameter of the stepped structure *w*_6_; (**h**) Effects of varying the parameter of *l*_7_.

As shown in Figure 3b, on the basis of the prototype, a section of the bending structure at the end is removed to reduce the inductive resistance of the tag antenna. Next, a non-uniform stepped structure is introduced on the radiation arm, which changes the current path through the stepped physical boundary. This compensates for some of the electrical length lost due to the removal of bend section. Based on this theory, the impedance of the tag antenna can be tuned by adjusting the geometric parameters (*l*_3_, *l*_4_, l_5_) of the stepped structure, as shown in Figure A1a–c. Although the tag antenna is more adjustable at this time, the impedance of the tag antenna at 927 MHz is capacitive. It cannot achieve conjugate matching with the capacitive R6 chip.

On the basis of the trapezoidal folding dipole, a T-match is ultimately introduced to reduce the capacitance of the tag antenna and increase inductive reactance. From the perspective of the physical mechanism of the equivalent circuit, the T-shaped shorting branch is connected across the feed point. Essentially, this can be viewed as an adjustable inductive branch connected in parallel with the resonant circuit of the tag antenna. This parallel inductance shifts the input reactance of the tag antenna from negative (capacitive) to positive (inductive). By adjusting the parameters of the T match branch (*w*_1_, *w*_2_, *l*_6_), as shown in Figure A1d–f, the impedance of the tag antenna can also be adjusted.

### 3.2. Analysis of Tag Antenna Based on Characteristic Mode

The characteristic mode theory can directly reveal the intrinsic resonance properties of the antenna without considering the specific form of the feed structure [24]. By analyzing the current distribution of different modes, the primary radiation mode of the antenna can be determined.

Therefore, on electrically small antennas such as miniaturized tag antennas, the characteristic mode theory can be used to analyze different current modes in the tag structure. Based on the distribution of these currents, the primary mode that contributes the most to radiation can be identified. In the design of this tag, the characteristic mode theory is mainly used to analyze the contribution of the structural design of the folding dipole arm to the radiation mode of the tag antenna.

**Figure 4:**
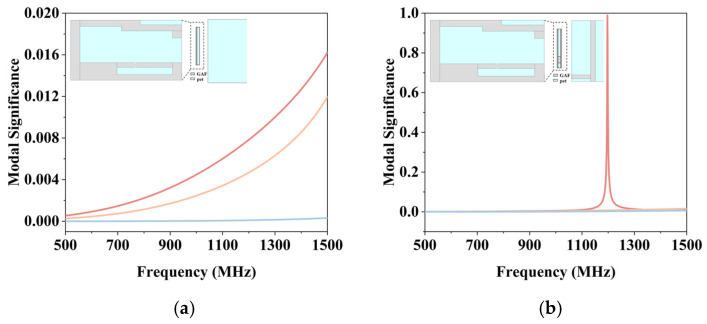
Modal significances of the tag antenna: (**a**) Modal significances in the absence of folding arm structure; (**b**) Modal significances in the case of folding arm structure.

**Figure 5:**
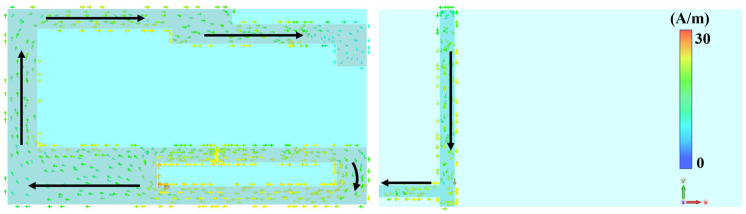
Current distribution of the tag antenna at 927 MHz.

**Figure 6:**
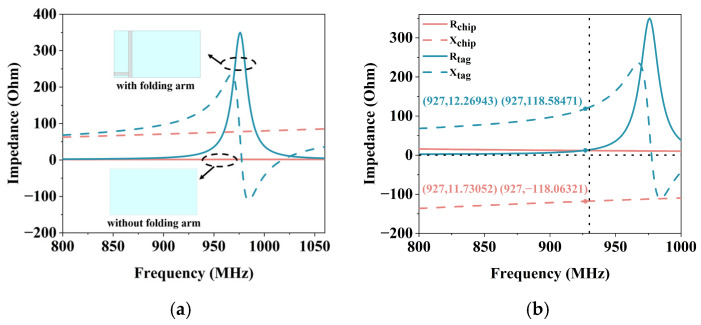
The impedance curve of the tag antenna: (**a**) Impedance comparison with or without folding arm structure; (**b**) Comparison of the proposed tag antenna and chip impedance.

Analysis of the characteristic model shows that the folded dipole arms do not simply serve as ground planes, but participate in the radiation of the tag antenna. As shown in Figure 4a, the modal significances corresponding to the three modes of the tag antenna are presented when the folding arm is removed, and only the upper-layer structure is retained. It can be seen that when only the upper bending arm structure remains, the maximum modal significance of the tag antenna is only 0.016. This indicates that the tag antenna does not resonate in the UHF band and essentially lacks the ability to radiate electromagnetic waves. As shown in Figure 4b, the modal significances corresponding to the three modes of the tag antenna after the addition of the folding arm are presented. It can be seen that the tag antenna exhibits the modal significance that approaches 1 at 1200 MHz. This indicates that the tag antenna resonates at this frequency, which is its primary radiation mode. It is worth noting that while the CMA reveals a primary structural self-resonance at 1200 MHz, where the intrinsic reactance of the complete antenna structure approaches zero, the tag actually operates at 927 MHz. This frequency offset is a fundamental requirement of passive RFID design. Because the chip is highly capacitive, the antenna must conjugate-match it by operating in an off-resonance, strongly inductive region. The antenna surface current distribution is shown in Figure 5. At 927 MHz, the strong currents are primarily concentrated in the T match structure and the folded region connecting the top and bottom layers. Meanwhile, the current at the end of the stepped radiating arms gradually weakens. The designed tag antenna is miniaturized from the structural design level by using the method of space folding. It combines the operating principle of an inverted-L antenna with a dipole; a T match structure is used to dominate the impedance tuning of the tag antenna. Figure 6a shows a comparison of the impedance of the tag antenna with and without the folding arm. Miniaturization is achieved by folding one arm of the dipole to the back, and together with the stepped dipole arm, it forms the main radiation mode of the antenna. Figure 6b shows the simulated input impedance of the proposed tag antenna. The impedance of the tag antenna is 12.27 + j118.58 Ω at 927 MHz, which almost conjugates the impedance of the chip to 11.73 − j118.06 Ω, indicating that the designed tag antenna structure is reasonable.

### 3.3. Antenna Characteristics

**Figure 7:**
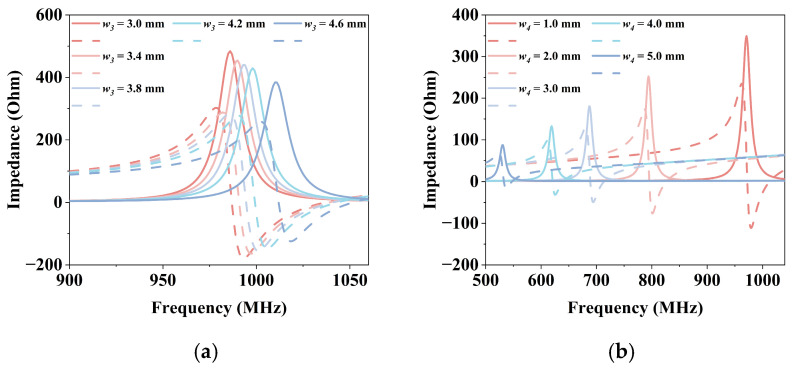
Parametric analysis of the folding arm: (**a**) Effects of varying the length of the conductor branch *w*_3_; (**b**) Effects of varying the width of the folding arm *w*_4_.

The parameter tuning of the folding arm is performed. The length of the conductor branch *w*_3_ will affect the resonant frequency of the antenna. As shown in Figure 7a, the resonant frequency can be tuned from 986 MHz to 1010 MHz by adjusting the length of the conductor branch. As one arm of the dipole, the folding arm participates in the radiation of the tag antenna. Its width *w*_4_ change can be seen as a way to lengthen or shorten the current path. As shown in Figure 7b, adjusting the width of the folding arm affects the resonant frequency and impedance of the antenna. The span change of the resonant frequency of the tag antenna from 531 MHz to 971 MHz can be realized, and the impedance becomes smaller while the resonant frequency shifts to the low frequency. As can be seen, these two parameters can be used in combination to adjust impedance and resonant frequency fairly effectively to achieve impedance matching with multiple chips.

**Figure 8:**
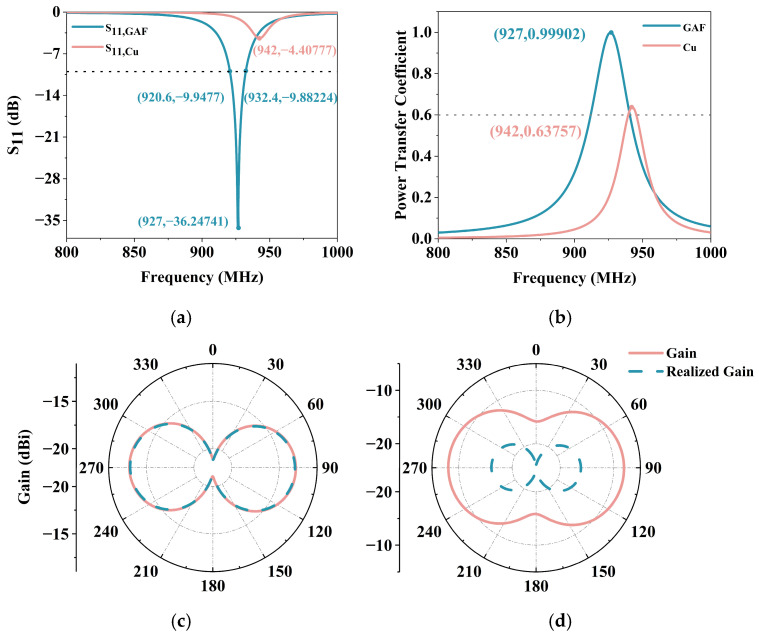
Performance of the tag antennas: (**a**) S_11_ of the GAF and Cu tag antennas; (**b**) τ of the GAF and Cu tag antennas; (**c**) Gain of GAF tag antenna; (**d**) Gain of Cu tag antenna.

The reflection coefficient (S_11_) of the tag antenna is calculated using $S_{11}={(Z}_{a}-Z_{c}^{*})/(Z_{a}+Z_{c})$ relative to the complex chip impedance. Where $Z_{c}=R_{c}+jX_{c}$ and $Z_{a}=R_{a}+jX_{a}$ are chip impedance and antenna impedance, respectively. The $Z_{a}$ is obtained by defining a discrete edge port in the simulation model to simulate ideal electrical contact. Then in the schematic environment, set the external port to import the chip impedance to calculate S_11_. The S_11_ curve of the tag antenna with frequency is shown in Figure 8a. It is clearly visible from the figure that the S_11_ value of GAF tag is as low as −36.25 dB at 927 MHz, while the copper tag is only −4.41 dB at 942 MHz. This indicates that the antenna exhibits excellent impedance matching and minimal energy reflection at this frequency.

Meanwhile, the −10 dB bandwidth of this antenna is about 920.6 MHz to 932.4 MHz, and its operating frequency band is highly suitable for typical UHF RFID applications. The quality of the impedance matching between the tag antenna and the chip can be evaluated by the power transfer coefficient τ, which represents the proportion of power actually transferred to the chip. It can be calculated by using $\tau =(4R_{a}R_{c}){/\left|Z_{a}+Z_{c}\right|}^{2}$, 0 ≤ τ ≤ 1. As shown in Figure 8b, the τ of the GAF tag reaches 99.9% at 927 MHz, which efficiently transmits most of the RF energy received to the chip and τ of the copper tag is only 63.8%.

To evaluate the radiation performance, the simulated gain (G), realized gain (G_realized_), radiation efficiency ($\eta_{\mathrm{rad}}$), and total efficiency ($\eta_{\mathrm{tot}}$) of the proposed GAF tag are extracted, as summarized in Table 2. As shown in Figure 8c, the simulated gain and realization gain of the GAF tag antenna are almost identical. The excellent conjugate matching achieved by the GAF tag is confirmed. Since the mismatch loss is negligible, the total efficiency (3.96%) is nearly equal to its radiative efficiency (3.97%). Conversely, as shown in Figure 8d, a significant gap exists between the gain and realized gain of the copper tag. Although the copper tag exhibits a higher radiation efficiency (10.1%) due to the superior conductivity, the severe impedance mismatch (τ = 0.638) degrades its total efficiency to 6.44%. More importantly, copper RFID antenna does not possess the advantages such as flexibility and corrosion resistance.

**Figure 9:**
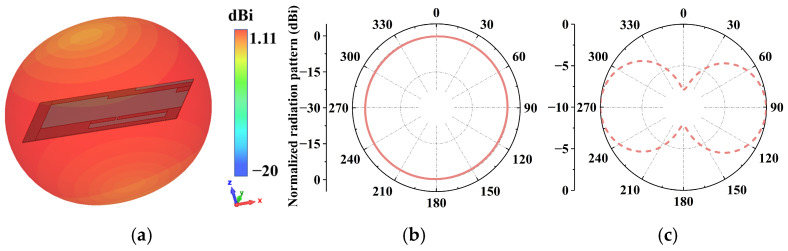
Simulated radiation patterns of the proposed tag antenna at 927 MHz: (**a**) Directional pattern of the tag antenna; (**b**) xy plane; (**c**) yz plane.

As shown in Figure 9a, the tag antenna exhibits typical dipole-like space radiation characteristics with good omnidirectional performance. The directional pattern in the xy plane in Figure 9b presents a perfect circle that transmits and receives signals from all angles of the plane uniformly and 360°. The directional pattern of the yz plane in Figure 9c exhibits a standard “8” curve, with the greatest radiation intensity at 90° and 270°.

### 3.4. Measurement Results and Analysis

**Figure 10:**
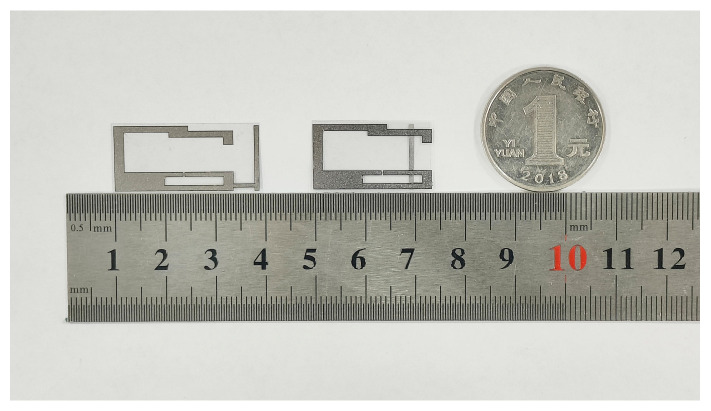
Prototype of the proposed tag antenna.

The GAF tag antenna processed by the laser engraving machine (LPKF Protolaser U4) has a clear pattern and regular edges, which meet the simulation and design expectations. The electrical interconnection between the chip and the antenna is established via conductive silver-paste dispensing, guaranteeing a reliable and effective contact. The actual tag antenna produced is shown in Figure 10, which has extremely compact dimensions. The first tag on the left is the original form without folding. Then fold the dipole arm along the T match structure to obtain the designed folded dipole tag antenna. The designed tag antenna uses GAF as the radiator material, which has excellent flexibility and corrosion resistance. Experimental tests were conducted to verify its mechanical stability and corrosion resistance in tag antenna applications.

**Figure 11:**
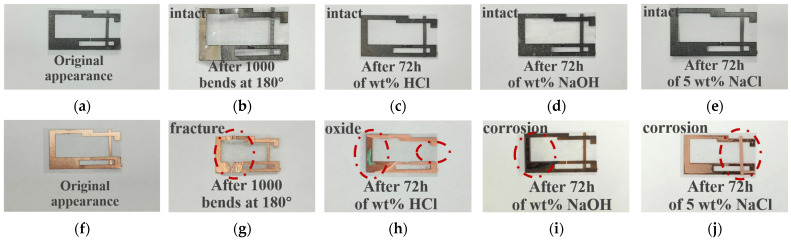
Comparison of bending durability and corrosion resistance between GAF tags and copper-tags: (**a**) Original appearance of the GAF tag; (**b**) After being 180° for 1000 cycles; (**c**) After 72 h 5 wt% HCl immersion; (**d**) After 72 h 5 wt% NaOH immersion; (**e**) After 72 h 5 wt% NaCl immersion; (**f**) Original appearance of the copper-tag; (**g**) After being 180° for 1000 cycles; (**h**) After 72 h 5 wt% HCl immersion; (**i**) After 72 h 5 wt% NaOH immersion; (**j**) After 72 h 5 wt% NaCl immersion.

**Figure 12:**
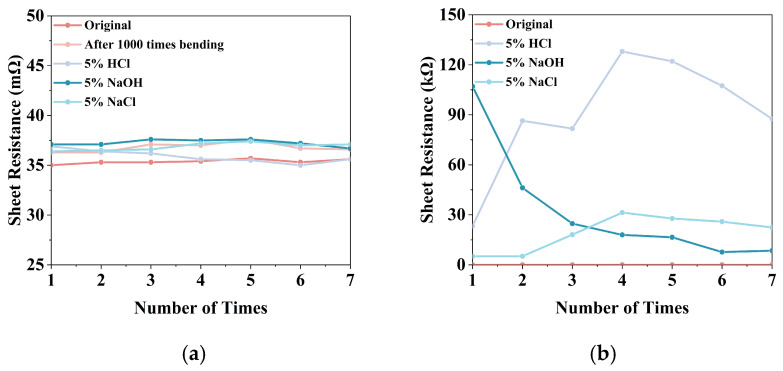
The sheet resistance of GAF and copper: (**a**) Sheet resistance of the original GAF compared to acidic, alkaline, saline exposure and after 1000 times bending; (**b**) Sheet resistance of the original copper compared to acidic, alkaline and saline exposure.

First, the tag antenna is subjected to repeated bending using a motor-driven sliding rail test platform. Set the slide rail speed to 5 mm/s, and perform a single bending cycle from 0° to 180° and back to 0°with a bending radius of R = 3 mm. As shown in Figure 11b, after 1000 bending cycles, the radiating structure of the GAF tag remains intact, and there is no mechanical damage such as cracks or delamination. The sheet resistance of the GAF tags showed remarkable stability, maintaining its original value of 3.5 × 10^−2^ Ω/sq with a variation of less than 0.2 mΩ/sq. As shown in Figure 12a, this result demonstrates that the GAF tag antenna exhibits good flexibility and mechanical stability. In contrast, the reference tag made of bare copper foil (thickness 20 ± 1 µm) fractured quickly during the bending process due to low mechanical toughness, as shown in Figure 11g. As comprehensively verified in our previous studies [25], GAF can endure over 200,000 extreme bending cycles with negligible DC resistance variation, whereas conventional copper foils typically fracture after merely a dozen cycles.

Next, the GAF and copper tag antennas are immersed in 5 wt% HCl, 5 wt% NaOH, and 5 wt% NaCl solutions for 72 h to evaluate the corrosion resistance of the tags. As shown in Figure 11c–e, after 72 h of corrosion treatment, the surface of the GAF tag maintains a smooth, dense, and continuous structure with no obvious corrosion, blistering, or oxidation spots.

Figure 12a shows that the GAF material maintains a sheet resistance of (3.5 ± 0.2) × 10^−2^ Ω/sq with no measurable degradation. In contrast, Figure 11h–j illustrate corrosion pits and oxide deposits on the bare copper reference tags, which compromise the continuity and integrity of the tag structure. Furthermore, four-probe measurements (Figure 12b) indicate that the sheet resistance of the corroded copper areas surged from an initial value of approximately 0.8 mΩ/sq to the kilo-ohm (kΩ/sq) level, exhibiting extreme spatial non-uniformity. Additionally, severely rusted sections of the copper tag fractured entirely, resulting in a complete loss of DC conductivity in those regions. Such electrical degradation increases the ohmic loss and impedance mismatch, leading to the total functional failure of the copper tag. This indicates that the GAF tag has good mechanical stability and corrosion resistance.

Read Range is a relatively intuitive indicator used to evaluate the performance of RFID tags. UHF tags mainly rely on the principle of backscattering for passive communication. Based on the classic Friis transmission equation, the maximum reading range of a tag can be calculated in a free space environment without interference [26].

(1) $$d_{\max}=\frac{\lambda }{4\pi }\sqrt{\frac{\mathrm{EIRP}\cdot G_{\mathrm{tag}}\cdot \tau }{P_{\mathrm{th}}}}$$

where λ is the wavelength, EIRP represents the output power allowed by national regulations for RFID readers, such as 4 W in North America and 3.28 W in China. G_tag_ denotes the gain of the tag antenna, P_th_ represents the tag sensitivity, which is also a crucial factor influencing read performance.

**Figure 13:**
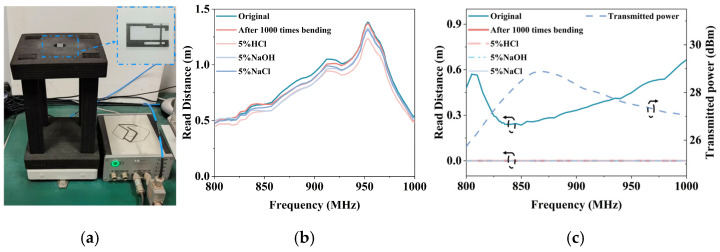
Read range test of the proposed tag antenna: (**a**) Measurement setup for measuring read range; (**b**) Read range of the original GAF tag compared to tags exposed to acidic, alkaline, saline and after 1000 times bending; (**c**) Read range of the Cu tag and the transmitted power of the reader reference tag.

The antenna is measured using the Voyantic Tagformance Pro test system shown in Figure 13a. Measurements are conducted in a controlled laboratory environment with no metals or reflectors within ≥1 m of the antenna to reduce environmental scattering. The tag is placed on a foam bracket at 30 cm from the 6.0 dBi line polarization antenna, with a polarization alignment deviation controlled to <10°. Path loss calibration was completed using a reference tag prior to testing. The transmitted power of the reader reference tag is shown in Figure 13c. Considering the equipment precision and environmental factors, the measurement uncertainty was estimated as ±0.5 dB.

To ensure the statistical reliability of the performance evaluations, a batch of five GAF tag samples is fabricated and tested. When the EIRP of the reader is 2.2 W, the maximum read range of the tag in the 800–1000 MHz is 1.38 m, with a standard deviation of ±0.06 m. Notably, the measured read range exhibits a certain reduction compared to the theoretical value of around 2 m calculated from the simulated gain using the Friis transmission equation [26]. This discrepancy is mainly caused by the manufacturing losses of the conductive silver-paste and measurement uncertainty, which is reasonable and within an acceptable range to a certain extent. Although the −10 dB impedance bandwidth is from 920.6 MHz to 932.4 MHz, the −20 dBm sensitivity of the Monza R6 chip allows the tag to harvest sufficient energy even under mismatch conditions. As a result, the measured readable bandwidth beyond the impedance bandwidth, covering the 800–1000 MHz range as demonstrated in Figure 13b. The reading range is measured after the tag underwent mechanical bending tests and corrosion tests in acid, alkali, and salt solutions. Measurements showed that the performance of the GAF tag antenna remained stable before and after the test, with no significant degradation. Importantly, the standard deviation of the read range remained bounded between ±0.03 m and ±0.06 m even after the mechanical bending and chemical corrosion tests. This confirms the excellent performance of the proposed GAF tag in maintaining stable electrical conductivity and radiation performance. As shown in Figure 13c, in contrast, due to poor impedance matching, the measured read range of the copper tag is lower than its theoretical value, reaching a maximum of only 0.66 m. Furthermore, after the bending and acid–alkali–salt corrosion tests, the copper tag antenna became completely unreadable.

In addition, Table 3 provides a comparative analysis of the proposed flexible miniaturized GAF RFID tags with other reporting tags. To objectively compare the proposed tag antenna, a normalized metric $k=d/(\sqrt{EIRP}\cdot S)$ is employed to characterize its space efficiency. Physically, dividing by $\sqrt{EIRP}$ normalizes the varying transmitted powers according to the Friis transmission equation, while dividing by the area S normalizes the performance against the physical footprint, enabling a fair evaluation of miniaturized tags. The comparison shows that the proposed tag antenna is capable of achieving a relatively long read range based on compact size.

## 4. Conclusions

A miniaturized flexible corrosion-resistant dipole tag antenna is proposed with a folding arm for UHF. The folding arm significantly miniaturizes antenna size by folding one arm of the dipole to the back. The folded arm itself does not occupy any additional surface area of the tag antenna, and together with the stepped dipole arm, it forms the main radiation mode of the antenna. Changing the size of the folding arm can also help adjust the input impedance and resonant frequency of the tag. In addition, the T-matching structure is used to embed the chip into the feeder without additional space. When the tag antenna is tested at EIRP = 2.2 W, the maximum read range achieved in the UHF band is 1.38 m. The designed tag antenna uses GAF as the radiator material, which has excellent flexibility and corrosion resistance. Comparison with a copper tag through bending and corrosion tests showed that the GAF tag antenna performed stably before and after bending and corrosion, with no significant deterioration.

## Figures and Tables

**Table 1:** Design parameters of the proposed tag antenna.

Parameters	Size (mm)	Parameters	Size (mm)	Parameters	Size (mm)
*l*_1_	13	*l*_7_	0.8	*w*_5_	2
*l*_2_	24	*l*_8_	2	*w*_6_	1.5
*l*_3_	9	*w*_1_	1	*w*_7_	0.8
*l*_4_	2	*w*_2_	12	*h*	0.08
*l*_5_	1	*w*_3_	4	*z*	0.025
*l*_6_	1.2	*w*_4_	1	*m*	0.3

**Table 2:** Performance comparison of GAF tag under the same geometry.

Tag	Frequency (MHz)	S_11_ (dB)	τ	$\eta_{\mathrm{rad}}$	$\eta_{\mathrm{tot}}$	G (dBi)	G_realized_ (dBi)
GAF	927	−36.25	0.999	3.97%	3.96%	−13.25	−13.25

**Table 3:** Comparative analysis between the proposed flexible GAF RFID tags and other works.

Ref.	Reader Power (EIRP)	Tag (mm^2^)	Electrical Size $(\lambda_{0}$)	Read Range (m)	$\frac{d}{\sqrt{EIRP}\cdot S}$ × 10^−3^ $\left(m/W^{1/2}\cdot {\mathrm{mm}}^{2}\right)$	Material	Corrosion Resistance	Flexibility
[20]	4 W	50 × 50	0.155 × 0.155	9.00	1.80	Copper	No	Yes
[27]	4 W	28 × 13	0.087 × 0.040	1.75	2.40	Copper	No	Yes
[28]	4 W	42 × 50	0.130 × 0.155	5.20	1.23	Aluminum	No	Yes
[29]	4 W	50 × 50	0.155 × 0.155	7.29	1.46	Aluminum	No	Yes
[30]	4 W	56 × 56	0.173 × 0.173	7.00	1.12	Copper	No	No
[31]	3.28 W	100 × 20	0.309 × 0.062	3.2	0.88	Graphene	Yes	Yes
This work	2.2 W	24 × 13	0.074 × 0.040	1.38	2.98	GAF	Yes	Yes

$k=\frac{d}{\sqrt{EIRP}\cdot S}$ is defined to characterize the read performance of the tag, where *S* denotes the tag area. $\lambda_{0}$ is the free-space wavelength at 927 MHz.

## Data Availability

The original contributions presented in this study are included in the article. Further inquiries can be directed to the corresponding authors.

## Appendix A

The impedance of the tag antenna is adjusted by adjusting the parameters of the geometric parameters of the stepped structure (*l*_3_, *l*_4_, l_5_) and the T match branch (*w*_1_, *w*_2_, *l*_6_) are as follows:
